# Sexual Relationship Typologies, Multilevel Determinants, and HIV Among Adolescent Mothers in Eastern and Southern Africa: A Multilevel Latent Class Analysis

**DOI:** 10.1007/s10461-025-05024-y

**Published:** 2026-01-16

**Authors:** Luwam T. Gebrekristos, Allison K. Groves, Marie C.D. Stoner, Alex Ezeh, Félice Lê-Scherban

**Affiliations:** 1 Department of Epidemiology and Biostatistics, Drexel University, 3215 Market Street, Philadelphia, PA 19140 USA; 2 Department of Community Health and Prevention, Drexel University, Philadelphia, PA USA; 3 Women’s Global Health Imperative, RTI International, Berkeley, CA USA

**Keywords:** HIV, Adolescent mothers, Multilevel latent class analysis, Sexual relationships, Eastern and southern africa, Multilevel drivers

## Abstract

In Eastern and Southern Africa (ESA), there is heterogeneity within adolescent mothers’ (AMs’) sexual relationships. However, existing studies focus on single relationship characteristics, even though these characteristics do not operate in isolation. Moreover, no studies have examined multilevel predictors of AMs’ relationships. Identifying and characterizing typologies by multilevel factors can inform targeted interventions to lower HIV risk. Data are from the Population-based HIV impact Assessment (PHIA) Project on AMs (15–19 years) from 9 ESA countries (*N* = 2,761). We conducted multilevel latent class analysis to identify relationship typologies at the AM and community (PHIA cluster) levels, multinomial logistic regression to characterize typologies by multilevel predictors, and multivariable generalized estimating equations (GEE) log-binomial regression to calculate prevalence ratios of HIV for relationship typologies. There were 3 typologies: *married with minimal transactional sex* (61%), *unmarried peer partnership* (30%), and *working and high transactional sex* (9%). Individual-level (i.e., age, school enrollment, low-wealth household, number of sexual partners) and community-level factors (i.e., urbanicity and proportion of low-wealth households) were associated with typologies. *Married with minimal transactional sex* was associated with a 51% lower HIV prevalence compared to the *unmarried peer partnership* (adjusting for multilevel factors). We also identified 2 PHIA cluster-level classes: *high age-disparate marriage* (49%) and *low marriage* (51%). Low-wealth communities had higher odds of being *high age-disparate marriage* communities. Results suggest that AMs’ relationships fall into 3 distinct typologies and 2 community-level classes. Further, HIV prevalence varies across relationship typologies. Utilizing tailored approaches could optimize interventions that reduce AMs’ HIV risk in ESA.

**Supplementary Information:**

The online version contains supplementary material available at 10.1007/s10461-025-05024-y.

## Introduction

Adolescent birth rates in Eastern and Southern Africa (ESA) are some of the highest rates worldwide [1], with over one-third of girls in ESA giving birth before age 20 [2]. Adolescent childbearing can negatively impact the health of adolescent mothers and their babies, and also negatively affect their social, economic and educational outcomes [3–9]. For example, mother-to-child transmission of HIV among adolescent mothers is higher than among older mothers [10, 11]. Further, adolescent mothers are at higher risk for HIV [12] and school dropout [3] than non-parenting adolescent girls.

Despite adolescent mothers’ heightened HIV risk in ESA [13], they are overlooked in HIV research [14]. This research gap has profound implications for existing interventions. Pregnant and postpartum adolescent girls may seek services from existing public health programs developed for adult mothers resulting in suboptimal antenatal attendance for adolescent mothers [15–17]. Specifically, adolescent mothers are less likely to initiate or complete standard mother-to-child transmission prevention programs compared to adult mothers [10, 18–20]. Moreover, current adolescent-friendly HIV prevention programs rarely account for the unique struggles adolescent mothers face [14].

Though adolescent mothers face high HIV risk, there is heterogeneity in HIV-related outcomes within this vulnerable population [10, 13, 18, 21]. Emerging evidence suggests that there is variability within adolescent mothers in HIV/STI risk [13, 21], and engagement in mother-to-child transmission prevention programs [10, 18]. Yet, the vast majority of research on adolescent mothers in ESA aggregates adolescent mothers with non-parenting girls or compares adolescent mothers’ HIV risk to other populations (i.e., nulligravida adolescent girls or adult mothers) [3, 13, 22–26]. While two studies have explored HIV/STI risk behaviors among adolescent mothers [21, 27], only one of the studies empirically examined heterogeneity of HIV/STI diagnoses, identifying intimate partner violence during pregnancy as a predictor of postpartum STI risk [21]. Given the variability in HIV risk among adolescent mothers and their poor adherence to existing HIV interventions, it is important to identify risk factors that lead some adolescent mothers to be at greater risk than other adolescent mothers.

While there is scant literature on HIV risk factors among adolescent mothers, studies indicate that power dynamics within their sexual relationships vary [22, 28] and these differences in their relationships may impact adolescent mothers’ HIV risk. Conceptually, different aspects within adolescent mothers’ sexual relationships may contribute to imbalanced power dynamics, which can increase HIV risk [29–31]. Specifically, having an older partner, engaging in transactional sex, or not having knowledge of their partner’s HIV status may influence adolescent mothers’ HIV/STI risk by decreasing their relationship power and subsequently, their ability to negotiate safer sex [21, 22, 27, 32–38], increasing their HIV risk. However, the association between marital status/cohabitation, relationship power and HIV risk has been inconsistent across studies. For instance, one latent class analysis found that a typology characterized by cohabiting with a partner and high relationship power was associated with higher rates of partner HIV testing and results sharing among adolescent girls and young women (AGYW) in Johannesburg [37]. Further, another study reported that married/living with a partner was linked to lower relationship power among AGYW residing in large cities in Kenya and South Africa [39]. In contrast, a third study found no association between marriage and HIV among adolescent girls across ESA [13]. These mixed findings may reflect differences in contextual norms shaped in part by geographical setting (e.g., urban, rural), the prevalence of marriage within study populations, or sample size. Further, given that adolescent motherhood often leads to school dropout [3], increasing their economic vulnerabilities, it is also important to consider the implication of financial power in their relationship. Adolescent mothers’ employment status can impact financial power and relationship power dynamics, which may also influence HIV-related behaviors (e.g., condom use) [40, 41]. Specifically, being unemployed may increase an adolescent mother’s reliance on her partner for financial support, limiting her power and decision-making within her sexual relationships, including her ability to negotiate safer sex, and increasing HIV risk [41].

A major limitation of the existing literature among adolescent mothers is that studies which examine the influence of power dynamics on sexual relationships typically focus on a single domain to capture relationship power, even though these domains do not operate in isolation. While multiple relationship characteristics simultaneously impact HIV risk, existing studies assess the association between a single relationship factor and an HIV-related outcome while controlling for other salient relationship characteristics [21]. Such variable-centered approaches (e.g., multivariable logistic regression) fail to capture the combination of factors that work together to predict such outcomes. For example, some married adolescent mothers may be in relationships that are protective against HIV (e.g., knowledgeable of partner’s HIV status and no transactional sex), whereas other married adolescent mothers are in relationships that heighten their HIV risk (e.g., older partner and being unemployed). Using a person-centered analytic approach (i.e., latent class analysis) to examine the distribution and intersection of these characteristics within adolescent mothers’ relationships is key to unpacking their risk. Given partners’ substantial influence on adolescent mothers’ sexual risk behaviors [27, 42], classifying adolescent mothers’ relationships into typologies will support in the identification of adolescent mothers who are most at-risk.

Latent class analysis (LCA) is a person-centered, model-based approach that is well-suited to identify relationship typologies by grouping adolescent mothers into distinct, mutually exclusive classes based on relationship characteristics [43]. Though other person-centered methods exist (e.g., traditional cluster analysis), those methods group individuals without a formal statistical model whereas LCA provides fit statistics and estimates class membership probabilities [44]. Two studies have used latent class analysis (LCA) to identify relationship typologies and examine how the co-occurrence of relationship characteristics influence HIV risk among AGYW in ESA [37, 45]. In one study, AGYW with out-of-school older partner had twice the risk of HIV infection compared to AGYW with a monogamous HIV-negative peer partner [45]. Another study found that AGYW with stable, empowered relationships with older less risky partners were more likely to complete the HIV self-test cascade than AGYW with shorter, empowered relationships with peer partners and shorter relationships with risky partners [37]. Although studies have applied LCA to identify relationship typologies and examine HIV risk among AGYW, sexual relationships differ by motherhood status [3, 13, 14]. As a result, typologies identified in the broader adolescent population may mask those specific to adolescent mothers, and the association with HIV may also differ in this subpopulation.

Moreover, in accordance with the socioecological framework (which states that multilevel factors influence health), adolescent mothers’ sexual relationship typologies may differ based on individual- and community-level factors [46]. For example, in a study of relationship typologies of AGYW, younger age was associated with a typology characterized by brief relationship duration and peer partners [37]. Further, not being in school [36], having multiple partners in the past year [47], and low household wealth [48] were associated with low relationship power-related characteristics (e.g., uninformed of partner’s HIV status). Further, existing studies in ESA identify urbanicity [49] and poverty [50] as structural determinants of imbalanced power dynamics or risky relationship characteristics among AGYW . And yet, no studies have examined whether individual- and community-level factors predict relationship typologies among adolescent mothers. Characterizing adolescent mothers’ relationship typologies by multilevel factors can inform targeted HIV prevention strategies.

To address these gaps, we used multilevel latent class analysis (MLCA) to identify relationship typologies (or latent classes) among adolescent mothers across 9 countries in ESA. MLCA extends the traditional LCA framework to account for the nested structure of the data (adolescent mothers within communities). Further, we examined whether individual and community-level factors predict adolescent mother’s relationship typology and whether adolescent mother’s relationship typology was associated with positive HIV diagnosis.

## Methods

### Data

The cross-sectional, population-based data that were used in this secondary analysis study were drawn from the Population-based HIV Impact Assessment (PHIA) Project. Our analytic sample included participants from 9 countries: Eswatini (2016–2017), Kenya (2018–2019), Lesotho (2016–2017), Malawi (2015–2016), Namibia (2017), Tanzania (2016–2017), Uganda (2016–2017), Zambia(2016), and Zimbabwe (2015–2016) [51–58]; these countries are characterized by high adolescent birth rates and HIV prevalence among AGYW [2, 59–61].

PHIA collects HIV-related and sociodemographic data using a two-stage stratified cluster sampling design. The sampling frame consists of all households in the country, based on each country’s census. In the first stage, clusters (hereafter referred to as communities) are randomly selected, stratified by geographic location (urban versus rural). Households are then randomly selected within the selected communities. Data were collected by trained staff via computer-assisted personal interviews with all individual household members (aged 80 years or younger) who were willing and able to provide consent (≤ 17-year-olds needed parental consent and provide assent). Emancipated minors (e.g., adolescents with children) were conferred with the rights of adulthood and therefore did not require parental consent. For confidentiality, PHIA geocodes communities’ locations instead of respondents’ homes [62]. Study protocols were approved by the Centers for Disease Control and Prevention Institutional Review Board (IRB), the Columbia University Medical Center IRB, and relevant local regulatory bodies. The analyses were not pre-registered.

Our analytic sample was restricted to adolescents aged 15–19 years, who reported having a partner in the last 12 months and ever had a live birth. This sample included 2,761 adolescent mothers across 1,816 PHIA communities with an average of 1.5 adolescent mothers per community (ranging from 1 to 14).

### Measures

The outcome, HIV diagnosis, was defined as testing positive for HIV. To identify relationship typologies among adolescent mothers, we used 5 characteristics associated with relationship power in our analyses [21, 22, 27, 32–37, 39–41] informed of partner’s HIV status, age disparity, married/cohabiting, transactional sex, and employment status. These indicators are for the adolescent mother’s most recent partner within the last year. Informed of partner’s HIV status: were told partner’s status or partner received conclusive results when tested together versus did not know partner’s status or believed they knew even though they were not explicitly told. Age disparity: consistent with the existing literature on adolescent relationship dynamics, partner’s age 5 or more years older versus partner’s age less than 5 years older than their own age [35, 63–66]. Married or cohabiting: currently married or living with their partner versus single, widowed, divorced, or separated. Transactional sex: whether the adolescent mother entered the sexual partnership because the partner provided, or there was an expectation that the partner would provide, the adolescent mother with gifts, help pay for things, or help in other ways (single item, yes versus no). While the measure of transactional sex may not explicitly capture sex in exchange for gifts or money, it represents the adolescent mother’s perceived motivation for entering the relationship. We included this measure based on prior work which argues that transactional sex is sexual relationships motivated by the underlying expectation that sex will be exchanged for money or gifts [67]. Employment status was defined as self-report of having worked versus has not worked in the last 12 months. Further details about the measurement of relationship characteristics are available in the supplementary file.

Selected covariates were chosen based on empirical literature. Individual-level covariates that were hypothesized to impact relationship typologies included age, currently in school (yes versus no), number of sexual partners in the past year, and a binary measure of low household wealth [37, 45, 67]. PHIA calculates a score of household wealth using principal components analysis on household’s ownership of selected assets (e.g., toilet facilities, electricity). Scores are divided by PHIA into wealth quintiles with “1” being lowest and “5” being the highest. In this current study, those who were in the lowest quintile were coded “1,” and otherwise were coded as “0.” Community-level covariates included the PHIA urbanicity classification (urban versus rural), and the percentage of households in the PHIA community who were in the lowest wealth quintile (includes households without an adolescent mother).

### Analysis

Latent class analysis (LCA) is an exploratory statistical method that identifies latent classes, or relationship typologies, based on patterns in adolescent mothers’ responses to the measured relationship indicators [44]. For each adolescent mother, the probability of being in each latent class is dependent on her response pattern [68]. If there are 4 latent classes, then each adolescent mother would have 4 membership probabilities. LCA assigns adolescent mothers to the latent class for which they have the highest membership probability.

An assumption of LCA is that the observations are independent of one another. Given that study participants are nested within PHIA communities, this independence assumption is violated. Multilevel LCA (MLCA) extends traditional LCA and accounts for the nested structure by identifying latent classes for adolescent mothers (level-1) and PHIA communities (level-2). Using the non-parametric approach in MLCA, latent classes at level-1 are estimated first, then C − 1 random means from the level-1 solution (where C is the number of level-1 latent classes) are used as indicators to estimate level-2 latent classes [69]. For example, if a 2-class solution is identified for level-1, one random mean would signify the log-odds of belonging to level-2 class 1 versus class 2, allowing for the log-odds to vary across the PHIA communities [69]. In using random means to define level-2 latent classes, PHIA communities are grouped based on their distribution of level-1 class memberships. PHIA communities are assigned to the level-2 latent class for which they have the highest membership probability.

Our MLCA occurred in two stages: (1) identify the number of level-1 and level-2 latent classes and (2) assess whether covariates predicted class membership across both levels. Since adolescent mothers and PHIA communities are probabilistically assigned to their latent classes, there is uncertainty in class assignment. This two-stage approach allowed us to examine the associations between latent classes and predictors while accounting for measurement errors related to uncertainty in class assignment [70]. All models in the two-stage approach were estimated using Mplus 8 [71].

- *Stage 1: Identify the number of latent classes.*
- To estimate the MLCA models, we used 5 relationship power indicators: informed of partner HIV status, age disparity, married or cohabiting, transactional sex, and employment status. We used full-information maximum likelihood (FIML) estimation with robust standard errors, allowing for missing data and accounting for the complex survey design.
  Following recommended practice, we used an iterative stepwise approach to identify the best-fitting model [69, 72]. First, we ignored the nested structure of the data by conducting a series of traditional LCA models to select the number of level-1 latent classes. Next, we conducted a series of MLCA using the best-fitting model from the previous step to select the number of level-2 latent classes. After selecting the number of level-2 latent classes, we assessed whether the number of level-1 latent classes changed by conducting a final series of MLCA using the selected number of level-2 latent classes to identify the number of level-1 latent classes. Model selection was guided by the Bayesian Information Criterion (BIC), a preferred measure for MLCA model selection, and interpretability of latent classes [72, 73]. Lower BIC values signify better model fit.
- *Stage 2: Assess whether covariates predict class membership.*
- Once a final model was selected, we included individual- and PHIA community-level covariates to predict level-1 class membership and PHIA community-level covariates to predictlevel-2 class membership. We used multinomial logistic regression to examine the associations between covariates and relationship typologies.

To calculate prevalence ratios of HIV for relationship typologies, we used generalized estimating equations (GEE) log-binomial regression with an exchangeable working correlation matrix and robust standard errors to account for clustering at the PHIA community-level. All pairwise typology comparisons were estimated to assess how each typology differs from the other typologies. In regression analyses, we excluded adolescent mothers with missing data on covariates and/or outcome (9.5%), yielding a sample of 2,626. Multicollinearity was not an issue for covariates based on variance inflation factors being < 10 (see supplementary file). GEE models were conducted in R [74].

## Results

Table 1 presents the descriptive statistics for the study sample. HIV prevalence was 4.2% among all adolescent mothers. The average age of participants was 18.2 years. Less than one in ten adolescent mothers were in school (8.3%) and one-third were from households with low wealth (31.0%). Approximately one-half of the sample was in an age-disparate relationship (51.2%). Nearly two-thirds were married or cohabitating with their partner (60.4%). Less than one in five reported transactional sex (16.8%), more than one-half were informed of their partner’s HIV status (55.0%), and less than one in four were working in the past year (22.7%). Nearly three-quarters of PHIA communities were in rural settings (72.2%). Eswatini had the fewest PHIA communities (85; 4.7%), and Uganda had the highest (312; 17.2%).

**Table 1:** Characteristics of study sample (*N* = 2,761)

	Overall
HIV diagnosis, n (%)	
Positive	117 (4.2%)
Negative	2,512 (91.0%)
Missing	132 (4.8%)
Individual Covariates	
Age, mean (sd)	18.2 (0.9)
Currently in school, n (%)	
Yes	230 (8.3%)
No	2328 (84.3%)
Missing	203 (7.4%)
Low household wealth, n (%)	
Yes	856 (31.0%)
No	1902 (68.9%)
Missing	3 (0.1%)
Number of partners in the past year, n(%)	
More than 1 partner	186 (6.7%)
1 partner	2575 (93.3%)
Country, n (%)	
Eswatini	105 (3.8%)
Kenya	281 (10.2%)
Lesotho	118 (4.3%)
Malawi	306 (11.1%)
Namibia	212 (7.7%)
Tanzania	544 (19.7%)
Uganda	539 (19.5%)
Zambia	362 (13.1%)
Zimbabwe	294 (10.6%)
Relationship Indicators	
Age disparate relationship, n (%)	
Partner ≥ 5 years older	1415 (51.2%)
Partner < 5 years older	1236 (44.8%)
Missing	110 (4.0%)
Marital status, n (%)	
Married or cohabiting	1668 (60.4%)
Not married or cohabiting	1085 (39.3%)
Missing	8 (0.3%)
Reported transactional sex, n (%)	
Yes	463 (16.8%)
No	2291 (83.0%)
Missing	7 (0.3%)
Informed of partner’s HIV status, n (%)	
Yes	1519 (55.0%)
No	1238 (44.8%)
Missing	4 (0.1%)
Worked in the past year, n (%)	
Yes	626 (22.7%)
No	2134 (77.3%)
Missing	1 (0.0%)
	*N* = 1,816
	Mean (SD) or N (%)
PHIA Community Covariates	
Community classification, n (%)	
Urban	505 (27.8%)
Rural	1311 (72.2%)
Proportion of households with low wealth, mean (sd)	24.5 (25.0)
Country, n (%)	
Eswatini	85 (4.7%)
Kenya	222 (12.2%)
Lesotho	97 (5.3%)
Malawi	212 (11.7%)
Namibia	158 (8.7%)
Tanzania	285 (15.7%)
Uganda	312 (17.2%)
Zambia	237 (13.1%)
Zimbabwe	208 (11.5%)

### Identifying Latent Classes

**Fig. 1:**
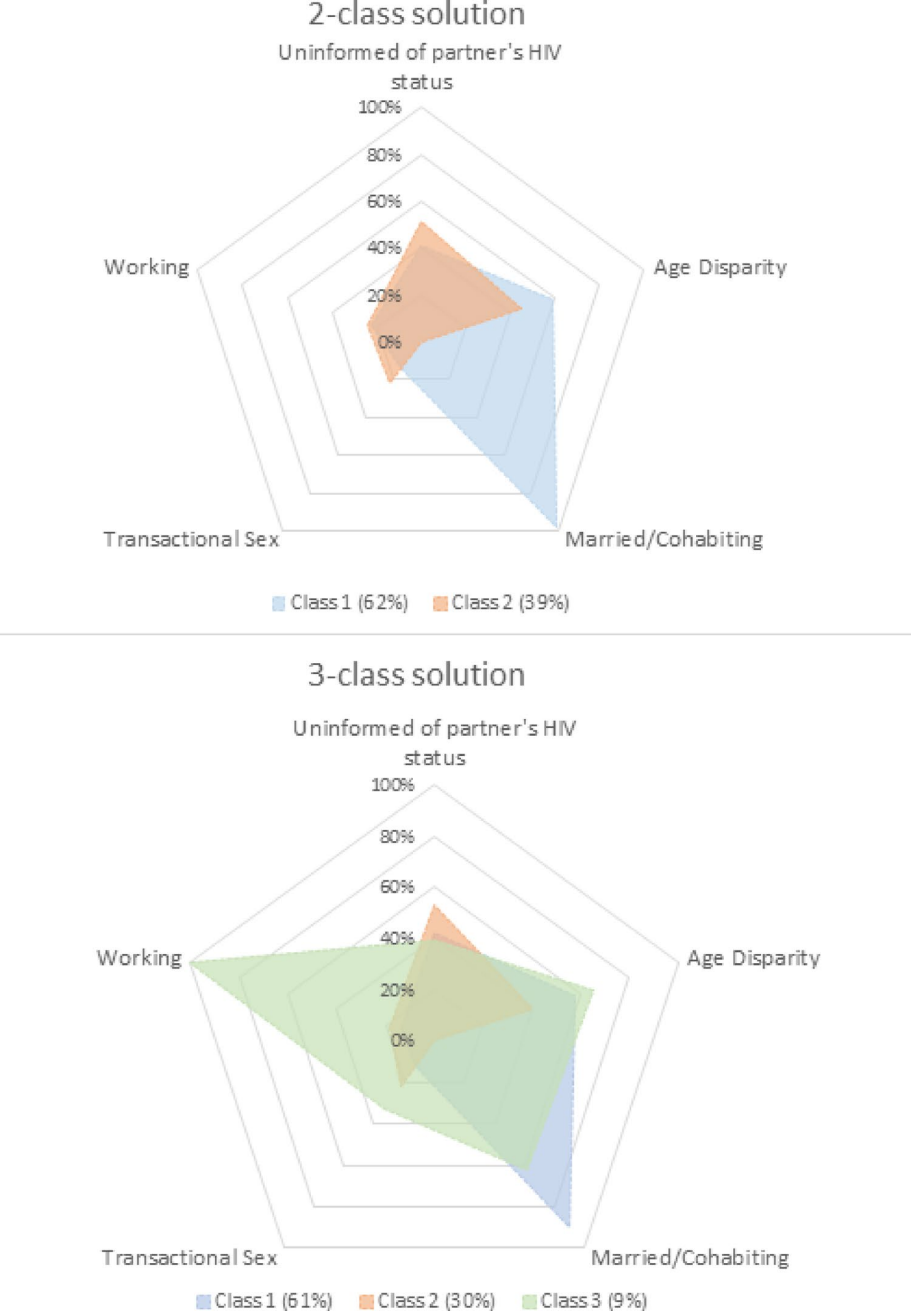
Traditional Latent Class Analysis 2-class and 3-class solutions

In the series of traditional LCA models, the 2-class solution appeared to be the preferred choice, as indicated by the lowest BIC (Table 2). However, the 2-class solution showed only marginally better model fit compared to the 3-class solution (BIC: 16,588.64 versus 16,611.09, respectively) and the 3-class solution provided more insightful interpretation of the latent classes (i.e., typologies). In the 2-class solution (Fig. 1), Class 1 and Class 2 had comparable probabilities for informed of partner’s HIV status, transactional sex and working. With the 3-class solution, there were large differences in the relationship indicators creating distinct latent classes. Given our interest in identifying and characterizing distinct relationship typologies among adolescent mothers, we prioritized the interpretability of our findings over the marginal improvement in model fit and selected the 3-class solution.

**Table 2:** Fit indices for traditional latent class analysis

Classes	Log likehood	AIC	BIC	ssBIC	BLRT *p*-value
1	−8299.13	16,608.27	16,637.88	16,622.00	–
2	−8250.74	16,523.48	16,588.64	16,553.686	< 0.0001
3	−8238.2	16,510.39	16,611.09	16,557.073	0.006
4	−8231.08	16,508.15	16,644.39	16,571.308	0.10
5	−8228.61	16,515.23	16,687.00	16,594.86	0.69

*AIC* Akaike Information Criterion, *BIC* Bayesian Information Criterion, *ssBIC* Sample sized adjusted Bayesian Information Criterion, *BLRT* Bootstrapped Likelihood Ratio Test

**Fig. 2:**
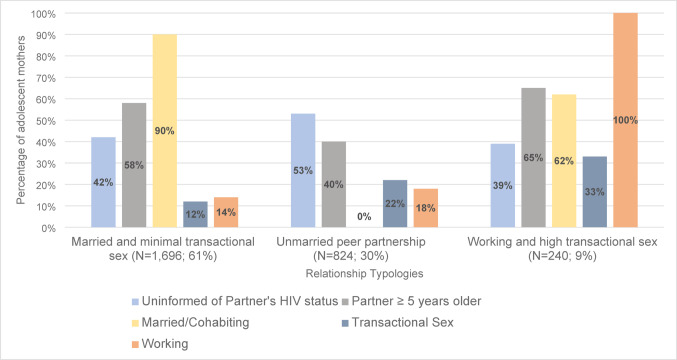
Describing Level-1 3-class solution by Relationship Indicators. Fig. 2 shows the conditional probabilities of endorsing each relationship characteristic within the identified level-1 latent classes. These probabilities are derived from the 3-class solution using multilevel latent class analysis

Typologies were defined based on the predominant characteristics within each typology. The largest typology (61% of adolescent mothers) was labeled as *married with minimal transactional sex* to capture the high occurrences of marriage or cohabitation (90% of adolescent mothers in this typology) and the low occurrence of transactional sex (12%; Fig. 2). Further, this typology is also characterized by high prevalences of age-disparate relationships (58%) and not working in the past year (only 14% worked in the past year). *Unmarried peer partnership*, the second largest typology, comprised of 30% of the sample and all were unmarried and not cohabiting. This typology is also characterized by peer partnerships (60%), uninformed of partner’s HIV status (53%), and not working in the past year (only 18% worked in the past year). The smallest typology (9%) was designated as *working and high transactional sex* as all adolescent mothers in this typology worked in the past year. Majority of adolescent mothers in this typology were married or cohabitating with their partners (62%) and had older partners (65%). Further, this typology had the highest occurrence of transactional sex compared to the other typologies (33% versus 12% and 22%, respectively). Taken together, *married with minimal transitional sex* and *working and high transactional sex typologies* reflect predominantly married or cohabiting adolescent mothers and age disparate relationships who differ in employment status and transactional sex. Whereas *unmarried peer partnership* typology represents unmarried adolescent mothers in peer relationships with lower awareness of partner’s HIV status.

Based on BIC values for the MLCA models, the 2-class solution had the best model fit compared to higher class solutions (supplementary file, model 1). Since increasing the number of classes did not enhance the interpretability of level-2 classes, we selected the 2-class solution for level-2. In re-estimating level-1 classes based on the level-2 solution (2 classes), we maintained the 3-class solution for level-1 based on interpretation of latent classes (supplementary file, model 2).

**Fig. 3:**
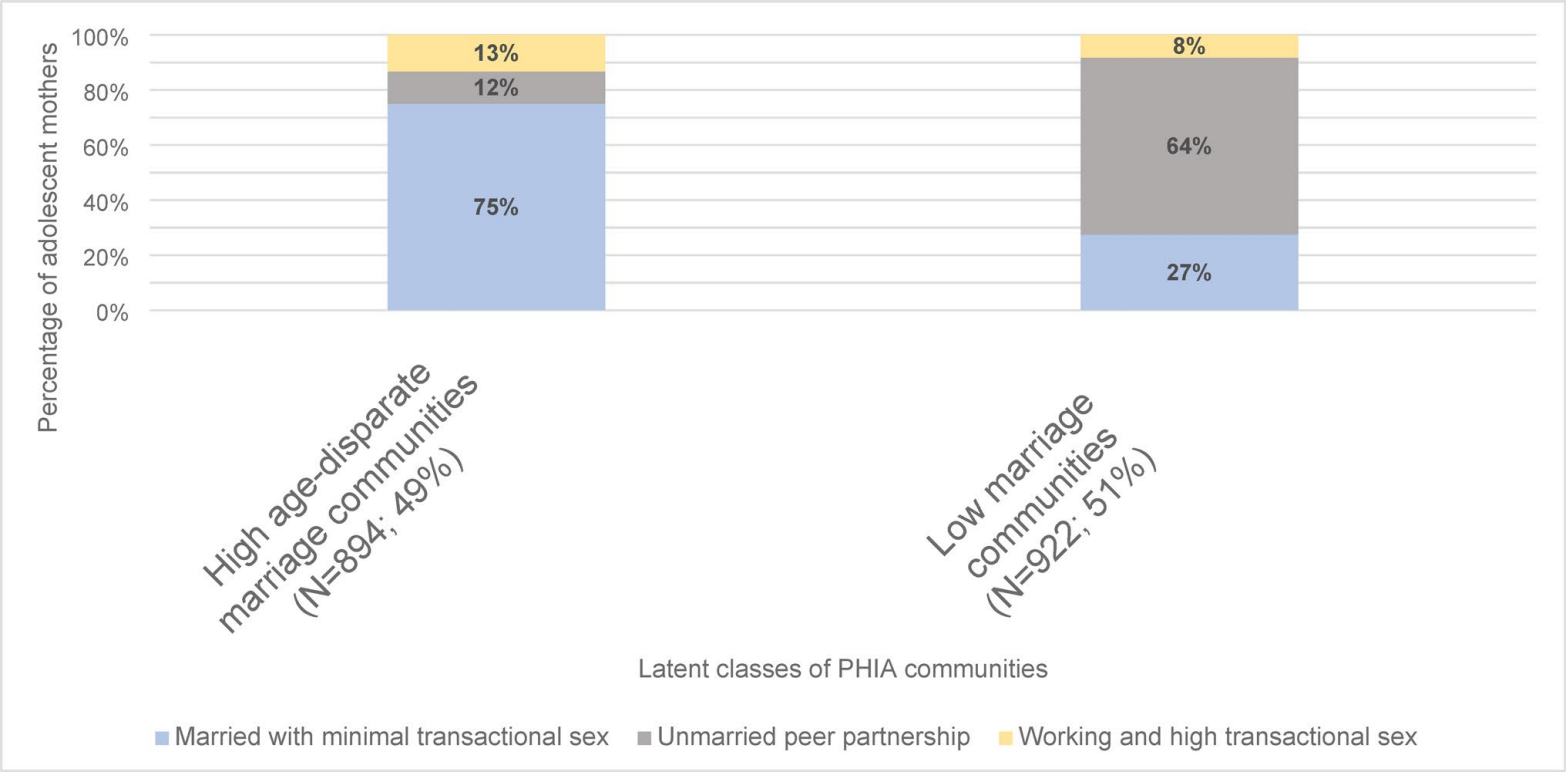
Multilevel latent class analysis Level-2, 2-class solution. Fig. 3 displays the conditional probability of membership in each level-1 latent class (relationship typology) within each level-2 latent class (PHIA community). These probabilities reflect how likely adolescent mothers in a given level-2 class belong to each of the identified level-1 classes

For level-2, PHIA communities were evenly split into two classes (Fig. 3). The first class, *high age-disparate marriage*, included PHIA communities (*n* = 894 or 49%) with high prevalence of level-1 *married with minimal transactional sex* adolescent mothers. In the second class, *low marriage*, PHIA communities (*n* = 922 or 51%) had a high number of *unmarried peer partnership* adolescent mothers.

### Predictors of Latent Classes

Table 3 shows prediction of level-1 class membership by covariates using multinomial logistic regression. Though the inclusion of covariates changed class sizes, the interpretation of classes remained the same. Model 1 includes level-1 predictors. Compared to being in the *unmarried peer partnership* typology, the odds of an adolescent mother being in the *married with minimal transactional sex* typology was significantly higher at older ages (per year, AOR: 1.39; 95% CI: 1.25–1.55) and if she was in a low-wealth household (AOR: 1.69; 95% CI: 1.35–2.11). Further, the odds of an adolescent mother being in the *married with minimal transactional sex* typology (compared to *unmarried peer partnership*) was significantly lower for those who were currently in school (AOR: 0.11; 95% CI: 0.07–0.18) and those with increased number of partners in the past year (AOR: 0.36; 95% CI: 0.25–0.51). Compared to being in the *unmarried peer partnership* typology, the odds of an adolescent mother being in the *working and high transactional sex* typology was significantly higher at older ages (per year, AOR: 1.33; 95% CI: 1.08–1.63) and significantly lower for those currently in school (AOR: 0.10; 95% CI: 0.03–0.36). Compared to *working and high transactional sex* typology, the odds of being in the *married with minimal transactional sex* typology was lower with increased number of partners in the past year (AOR: 0.32; 95% CI: 0.21–0.48).

**Table 3:** Multilevel latent class analysis with Level-1 and Level-2 covariates (Level-1 class membership)

Predictors	Model 1^a^			Model 2^b^				
	Married with minimal transactional sex versus Unmarried peer partnership	Working and high transactional sex versus Unmarried peer partnership	Married with minimal transactional sex versus Working and high transactional sex	Married with minimal transactional sex versus Unmarried peer partnership		Working and high transactional sex versus Unmarried peer partnership	Married with minimal transactional sex versus Working and high transactional sex	
	AOR (95% CI)	AOR (95% CI)	AOR (95% CI)	AOR (95% CI)		AOR (95% CI)	AOR (95% CI)	
Age	1.39 (1.25, 1.55)***	1.33 (1.08, 1.63)**	1.05 (0.87, 1.27)	1.41 (1.27, 1.58)***		1.36 (1.1, 1.67)**	1.04 (0.86, 1.26)	
Currently in school	0.11 (0.07, 0.18)***	0.10 (0.03, 0.36)***	1.12 (0.3, 4.25)	0.11 (0.07, 0.18)***		0.09 (0.02, 0.4)**	1.25 (0.27, 5.71)	
Low-wealth household	1.69 (1.35, 2.11)***	1.49 (0.98, 2.28)	1.13 (0.79, 1.61)	1.20 (0.91, 1.57)		1.16 (0.65, 2.06)	1.03 (0.63, 1.69)	
Number of partners	0.36 (0.26, 0.51)***	1.14 (0.79, 1.66)	0.32 (0.21, 0.48)***	0.38 (0.27, 0.54)***		1.19 (0.81, 1.74)	0.32 (0.21, 0.49)***	
Urban community	–	–	–	0.71 (0.55, 0.91)**		0.73 (0.42, 1.26)	0.97 (0.59, 1.58)	
Proportion of households with low wealth (10-percentage point increase)	–	–	–	10.08 (10.02, 10.13)**		10.05 (9.93, 10.17)	10.03 (9.92, 10.14)	

*AOR *Adjusted Odds Ratio,* CI *Confidence Interval

a = Model 1includes level-1 covariates; b = Model 2 includes level-1 and level-2 covariates

* p <.05; **p <.01; *** p <.0001

Model 2 includes both level-1 and level-2 covariates as predictors of level-1 class membership. Inclusion of level-2 predictors attenuated estimate for low-wealth household. Controlling for individual-level covariates, the odds of an adolescent mother being in the *married with minimal transactional sex* versus *unmarried peer partnership* typology was lower for those living in urban communities than rural communities (AOR: 0.71; 95% CI: 0.55–0.91). Further, the odds of an adolescent mother being in the *married with minimal transactional sex* versus *unmarried peer partnership* typology was higher as proportion of low-wealth households increased (for a 10-point increase, AOR: 10.08; 95% CI: 10.02–10.13). Only level-2 covariates were included as predictors of level-2 latent class membership (Table 4). Compared to *low marriage* communities, the odds of being a *high age-disparate marriage* community were significantly higher as proportion of low-wealth households increased (for a 10-point increase, AOR: 10.10; 95% CI: 10.06–10.14). Level-2 class membership was not associated with living in an urban or rural community.

**Table 4:** Multilevel latent class analysis with Level-2 covariates (Level-2 class membership)

	High age-disparate marriage versus Low marriage
Predictor	OR (95% CI)
Urban community	0.78 (0.59, 1.03)
Proportion of households with low wealth (10-percentage point increase)	10.10 (10.06, 10.14)***

*OR* Odds Ratio, *CI* Confidence Interval

* p <.05; **p <.01; *** p <.0001

### Relationship Typologies and HIV

HIV prevalence was 2.9% for *married with minimal transactional sex*, 6.5% for *unmarried peer partnership*, and 4.3% for *working and high transactional sex* typologies (data not shown). Table 5 shows the prevalence ratios of bivariate and multivariable analyses of relationship typologies and HIV. In bivariate analysis, *married with minimal transactional sex* typology was associated with a 54% lower HIV prevalence compared to the *unmarried peer partnership* typology (Model 1: PR: 0.46; 95% CI: 0.32–0.68). Adjusting for individual and community-level covariates resulted in similar estimates (Model 2: PR: 0.49; 95% CI: 0.32–0.73). There was no evidence of a significant difference in HIV prevalence between *unmarried peer partnership* and *working and high transactional sex* typologies and *working and high transactional sex* and *married with minimal transactional sex* typologies (Models 1 and 2). In the adjusted model, attending school was associated with a 64% lower HIV prevalence compared to not attending school (Model 2: PR: 0.36; 95% CI: 0.15–0.87). Further, HIV prevalence was significantly higher with increased number of partners (per partner, PR: 1.52; 95% CI: 1.19–1.94).

**Table 5:** Unadjusted and adjusted prevalence ratios of positive HIV diagnosis

	Model 1		Model 2^a^	
	PR (95% CI)	*p*-value	PR (95% CI)	*p*-value
Married with minimal transactional sex *(reference group: Unmarried peer partnership)*	0.46 (0.32–0.68)	< 0.0001	0.49 (0.32–0.73)	0.0006
Working and high transactional sex *(reference group: Unmarried peer partnership)*	0.66 (0.35–1.24)	0.20	0.60 (0.31–1.15)	0.13
Married with minimal transactional sex *(reference group: Working and high transactional sex)*	0.70 (0.36–1.36)	0.30	0.81 (0.41–1.60)	0.54
Covariates				
Age	–	–	1.04 (0.85–1.28)	0.70
Currently in school *(reference group: currently not in school)*	–	–	0.36 (0.15–0.87)	0.02
Low household wealth *(reference group: Middle to High household wealth)*	–	–	1.32 (0.80–2.17)	0.28
Number of partners in the past year	–	–	1.52 (1.19–1.94)	0.0008
Proportion of households with low wealth	–	–	0.99 (0.98–1.00)	0.06
Urban community *(reference group: rural community)*	–	–	1.45 (0.92–2.28)	0.11

*PR* Prevalence Ratio, *CI* Confidence Interval

Pairwise comparisons of latent class membership were conducted using generalized estimating equations (GEE) log-binomial models, which account for clustering at the PHIA community-level.

^a^ Model 2 accounts for age, in school, low-wealth household, number of partners in last year, proportion of households with low wealth in PHIA community, urban versus rural PHIA community

## Discussion

In ESA, adolescent mothers face high, yet varied, HIV risk and diverse relationship dynamics, yet adolescent mothers’ relationships have been underexplored. The purpose of the study was to identify relationship typologies among adolescent mothers in ESA, examine whether individual- and community-level factors predict adolescent mother’s relationship typologies and whether adolescent mother’s relationship typologies were associated with positive HIV diagnosis. Results suggest that adolescent mothers’ relationships fall into three distinct typologies: *married with minimal transactional sex*, *unmarried peer partnership*, and *working and high transactional sex*. PHIA communities were equally split into two classes: *high age-disparate marriage* and *low marriage* communities. Further, *unmarried peer partnership* had higher HIV prevalence than *married with minimal transactional sex* typology.

Using MLCA, we found that individual-level factors, age, school enrollment, low-wealth household, and number of sexual partners, were associated with adolescent mothers’ relationship typologies. However, inclusion for community-level factors attenuated estimate for low-wealth households. This finding indicates that the association between low-wealth households and relationship typologies is explained by community-level factors. Living in an urban community and decreased proportion of low-wealth households were associated with having an *unmarried peer partnership* (compared to *married with minimal transactional sex* relationship).

Further, proportion of low wealth households predicted latent classes for communities. Communities with a higher proportion of low-wealth households had higher odds of being labeled as *high age-disparate marriage*. Proportion of low-wealth households’ findings are similar to the level-1 results, in which adolescent mothers differ in the likelihood of being in *unmarried peer partnership* and *married with minimal transactional sex* typologies based on their communities’ proportion of low-wealth households and urbanicity. Results suggest that adolescent mother’s relationship typology and the distribution of typologies within a PHIA community are influenced by the wealth of her community. In contrast, urbanicity did not explain level-2 class membership, meaning it likely does not play a meaningful role in shaping the broader structural or contextual patterns captured at level-2.

Although the highest HIV prevalence was observed in the *unmarried peer partnership* typology and the lowest in the *married with minimal transactional sex* typology, we cannot determine which specific relationship characteristic(s) are driving these differences. Rather, our latent class analysis illustrates how multiple relationship factors cluster together to shape HIV risk and that these typologies reveal patterns not apparent when factors are examined in isolation. Importantly, the association between typology and HIV prevalence does not imply that being married or being in an age-disparate relationship alone is protective. Adolescent mothers in the *married with minimal transactional sex* typology reported less transactional sex than those in *unmarried peer partnership* (12% versus 22%, respectively) and were more informed of their partner’s HIV status. These differences may reflect the broader social and economic marginalization faced by unmarried adolescent mothers [75–77], rather than an inherent protective effect of marriage. Low transactional sex, coupled with high marriage/cohabitation, may reflect that adolescent mothers in the *married with minimal transactional sex* typology are more likely to be in longer-term, stable relationships [78]. Such relationships may be characterized by higher social support [45]. This inferred relationship stability may also explain why this typology had higher reports of knowledge of partner’s HIV status, as longer-term relationships provide greater opportunities for communication and disclosure [22, 37]. For adolescent mothers in the *married with minimal transactional sex* typology, being married or cohabiting with partner and having strong communication with their partners, as evidenced by knowing one another’s HIV status, may imply more equitable relationship dynamics (even despite disparate ages), thus reducing their HIV risk. While age-disparate relationships have been associated with increased HIV risk in prior studies [22, 38, 48, 65, 79], none of these studies distinguished between married and unmarried AGYW. Our findings highlight that HIV vulnerability arises through the broader relationship context. Conversely, relationships characterized as *unmarried peer partnership* were younger and reported the highest prevalence of transactional sex and being uninformed of partner’s HIV status. This finding may reflect limited couples HIV testing and disclosure among the *unmarried peer partnership* typology, which serve as a critical entry point to HIV risk management [80]. It is possible that the combinations of relational risk factors including lack of formal partnership (unmarried or not cohabiting), transactional sex and uninformed of partner’s HIV status contribute to lower relationship power and elevated HIV prevalence among adolescent mothers in the *unmarried peer partnership* typology. Our findings emphasize the importance of considering multiple factors in combination rather than focusing on a single risk factor.

Relationship typologies for adolescent mothers may differ from relationship typologies for adolescent girls. That is, two studies in South Africa found different relationship typologies than we did [37, 45]. In a Johannesburg study, three relationship typologies were identified: (1) stable, empowered relationship with older partners, (2) shorter, empowered relationships with peer partners and (3) shorter relationships with risky partners [37]. The first class was characterized by living with an older partner and associated with higher rates of partner HIV testing, which is similar to *married with minimal transactional sex* typology. In a rural Mpumalanga Province study, five relationship typologies were identified: (1) monogamous HIV-negative peer partner, (2) one-time protected in-school peer partner, (3) anonymous out-of-school peer partner (4) out-of-school older partner and (5) cohabiting with children in-school peer partner [45]. Of the participants who had children with their partners, the majority of their relationships were labeled “monogamous HIV-negative peer partner” (66%) [45]. This finding differs greatly from our finding that 61% of adolescent mothers were in *married with minimal transactional sex* relationships in which the majority had an older partner. Given that the relationship indicators used in our study and other studies varied, differences in LCA findings are not surprising. Further, differences in findings could be attributed to differences in study sample and setting. Specifically, our findings are from multiple countries with different adolescent marriage rates [2]. Marriage before age 18 is less common in South Africa compared to other ESA countries [2]. While indicators varied across all studies, examining similarities and differences can provide insights into how adolescent mothers’ relationships and needs differ.

Results suggest several directions for future research. First, further research is needed to understand additional positive relationship indicators (e.g., partner support) that mitigate adolescent mothers’ HIV risk. Second, we identified classes at the individual and community level; future research should build on the current study to examine other community-level factors that impact adolescent mothers’ relationships and HIV outcomes like HIV testing ratesor community norms about adolescent marriage (e.g., adolescent marriage rates). While *married with minimal transactional sex* typology had the lowest HIV prevalence, it is possible that timing of marriage in relation to pregnancy (i.e., married before or after pregnancy) may influence relationship power and subsequently HIV risk. Further research should explore whether timing of marriage influences relationship dynamics and HIV risk. Finally, it is possible that types of relationship typologies differ by geographic contexts (urban versus rural). Future research should stratify analyses by urban–rural residence to examine whether distinct relationship typologies emerge across these settings.

Given all three typologies were characterized by imbalanced power dynamics (e.g. transactional sex, uninformed of partner’s HIV status), there are several intervention implications. For example, adolescent mothers in the *unmarried peer partnership* typology had higher HIV prevalence than those in *married with minimal transactional sex* typology and were most likely to be in school. While attending school was protective against HIV, *unmarried peer partnership* may encompass vulnerabilities (e.g., imbalanced power dynamic) that outweigh the benefits of school enrollment, resulting in elevated HIV prevalence among adolescent mothers in the *unmarried peer partnership* typology. Conversely, older adolescent mothers in the *married with minimal transactional sex* typology may have completed school and therefore not currently enrolled. Exploring typologies reveals insights that are not apparent when variables such as schooling, marital status, or age disparity are considered in isolation. These findings suggest that HIV prevention interventions that address these vulnerabilities (e.g., uninformed of partners’ HIV status) among unmarried adolescent mothers with peer partners could occur in school settings. While HIV prevalence was lower among *married with minimal transactional sex* typology, adolescent mothers in *married with minimal transactional sex* and *working and high transactional sex* relationships were less likely to be enrolled in school. Considering that attending school mitigates HIV risk [81, 82], interventions that support adolescent mothers’ return to school may aid in reducing their HIV risk. Further, adolescent mothers in *married with minimal transactional sex* and *working and high transactional sex* relationships tend to be married to or cohabiting with older partners and therefore negotiating safer sex may be more difficult [37]. Further, we do not know their fertility desires; therefore, adolescent mothers in *married with minimal transactional sex* and *working and high transactional sex* could be good candidates for pre-exposure prophylaxis (PrEP). Recognizing the lack of interventions that address the specific needs of adolescent mothers, further attention is needed to develop HIV prevention interventions for this population.

In addition to the intervention implications discussed above, there are policy implications that stem from our level-2 findings. Given approximately half of the sample were uninformed of their partner’s HIV status, policies that increase HIV testing and foster communication with sexual partners about HIV status are needed. Wealthier communities were more likely to be labeled as *low marriage* communities and therefore had a high occurrence of *unmarried peer partnership*s—adolescent mothers are in school, uninformed of partner’s HIV status and had higher HIV prevalence. School-based sexual education programs in these wealthier communities may be a suitable venue for interventions that include discussion on HIV testing and communicating with sexual partners about HIV status.

Our study has several limitations. With the cross-sectional nature of our analysis, we are unable to infer causality. Next, only a small subset of participants in the PHIA sample were asked about intimate partner violence (IPV), precluding our ability to examine IPV as an indicator of relationship power in our analyses. Though our included relationship indicators are highly correlated with IPV [33, 63, 83, 84], the absence of IPV in our relationship typologies has implications for designing relationship-specific HIV prevention interventions. For example, intervention strategies would need to engage with violence response and/or prevention if high IPV was a key feature of a relationship typology. Further, condom use was reported for the last sexual act in the PHIA surveys, rather than over a specific time period, which limits interpretation of consistent condom use within the relationship. The surveys did not include questions on relationship duration, relationship power, the number of sexual partners or concurrent partnerships of adolescent mothers’ partners and partners did not undergo HIV testing, limiting our ability to examine these relationship characteristics in our analyses. Finally, this study excluded adolescent mothers younger than 15 because they were not asked about their sexual or reproductive history in the larger study. Therefore, it is possible that our findings may not be generalizable to younger adolescent mothers. However, childbearing prior to age 15 is low in the region (< 4%) [2].

Nonetheless, this study has several strengths. Our study is the first to examine relationship typologies among adolescent mothers across 9 ESA countries. Using a multilevel person-centered approach, this study examined individual and community-level predictors of adolescent mothers’ relationship typologies. Another methodological strength is we accounted for uncertainty in latent class membership in examining associations between predictors and relationship typologies.

With the elevated HIV burden among adolescent mothers in ESA, it is crucial to understand adolescent mothers’ relationships. This study broadens our understanding by examining the distribution and intersection of relationship indicators using a person-centered approach. Our findings indicate there are clear patterns in adolescent mothers’ relationships, with most adolescent mothers’ relationship falling in the *married with minimal transactional sex* relationship typology. Given that adolescence is a critical period for the establishment of sexual behaviors that impact current and future health outcomes, generating evidence using person-centered approaches support the development of interventions and policies will not only improve the health of adolescent mothers and their children, but also the future health of the region.

## Supplementary Information

Below is the link to the electronic supplementary material.

Supplementary Material 1

## Data Availability

The data are not publicly available. Requests to access the data should be sent to the Population-based HIV Impact Assessment Project.

## Code Availability

Code may be made available by contacting the corresponding author.

## References

[1] United Nations Department of Economic and Social Affairs, World Population P, Revision. Age-specific fertility rates by region, subregion and country, 1950–2100 (births per 1,000 women) Estimates 2024. https://population.un.org/wpp/Download/Standard/Fertility/

[2] Melesse DY, Cane RM, Mangombe A, Ijadunola MY, Manu A, Bamgboye E, et al. Inequalities in early marriage, childbearing and sexual debut among adolescents in sub-Saharan Africa. Reprod Health. 2021;18:117. 10.1186/s12978-021-01125-8. 34134718 10.1186/s12978-021-01125-8 PMC8210338

[3] Stoner MC, Rucinski KB, Edwards JK, Selin A, Hughes JP, Wang J, et al. The relationship between school dropout and pregnancy among adolescent girls and young women in South Africa: a HPTN 068 analysis. Health Educ Behav. 2019;46:559–68. 30819011 10.1177/1090198119831755 PMC6625926

[4] Toska E, Laurenzi CA, Roberts KJ, Cluver L, Sherr L. Adolescent mothers affected by HIV and their children: a scoping review of evidence and experiences from sub-Saharan Africa. Glob Public Health. 2020. 10.1080/17441692.2020.1775867. 32507031 10.1080/17441692.2020.1775867 PMC7578028

[5] Mee P, Fearon E, Hassan S, Hensen B, Acharya X, Rice BD, et al. The association between being currently in school and HIV prevalence among young women in nine eastern and southern African countries. PLoS One. 2018;13:e0198898. 10.1371/journal.pone.0198898. 29924827 10.1371/journal.pone.0198898 PMC6010266

[6] Ganchimeg T, Ota E, Morisaki N, Laopaiboon M, Lumbiganon P, Zhang J, et al. Pregnancy and childbirth outcomes among adolescent mothers: a World Health Organization multicountry study. BJOG: An Int J Obstet Gynaecol. 2014;121:40–8. 10.1111/1471-0528.12630. 10.1111/1471-0528.12630 24641534

[7] Rosenberg M, Pettifor A, Miller WC, Thirumurthy H, Emch M, Afolabi SA, et al. Relationship between school dropout and teen pregnancy among rural South African young women. Int J Epidemiol. 2015;44:928–36. 10.1093/ije/dyv007. 25716986 10.1093/ije/dyv007 PMC4521125

[8] Lloyd C, Mensch B. Marriage and childbirth as factors in school exit: an analysis of DHS data from Sub-Saharan Africa. Poverty, Gender, and Youth 2006.10.31899/pgy1.1013

[9] UNESCO. Early and unintended pregnancy and the education sector: evidence review and recommendations. n.d.

[10] Horwood C, Butler LM, Haskins L, Phakathi S, Rollins N. HIV-infected adolescent mothers and their infants: low coverage of HIV services and high risk of HIV transmission in KwaZulu-Natal, South Africa. PLoS One. 2013;8:e74568. 10.1371/journal.pone.0074568. 24073215 10.1371/journal.pone.0074568 PMC3779214

[11] Fatti G, Shaikh N, Eley B, Jackson D, Grimwood A. Adolescent and young pregnant women at increased risk of mother-to-child transmission of HIV and poorer maternal and infant health outcomes: a cohort study at public facilities in the Nelson Mandela Bay Metropolitan district, Eastern Cape, South Africa. S Afr Med J. 2014;104:874–80. 26042271 10.7196/samj.8207

[12] Christofides NJ, Jewkes RK, Dunkle KL, Nduna M, Shai NJ, Sterk C. Early adolescent pregnancy increases risk of incident HIV infection in the Eastern Cape, South Africa: a longitudinal study. J Int AIDS Soc. 2014;17:18585. 24650763 10.7448/IAS.17.1.18585 PMC3962027

[13] Groves AK, Gebrekristos LT, Smith PD, Stoebenau K, Stoner MC, Ameyan W, et al. Adolescent mothers in Eastern and Southern Africa: an overlooked and uniquely vulnerable subpopulation in the fight against HIV. J Adolesc Health. 2022;70:895–901. 10.1016/j.jadohealth.2021.12.012. 35172930 10.1016/j.jadohealth.2021.12.012 PMC9113251

[14] Groves AK, Maman S, Stankard PH, Gebrekristos LT, Amon JJ, Moodley D. Addressing the unique needs of adolescent mothers in the fight against HIV. J Int AIDS Soc. 2018;21:e25155. 29956491 10.1002/jia2.25155 PMC6024120

[15] Lawn J, Kerber K. Opportunities for Africa’s Newborns: practical data, policy and programmatic support for newborn care in Africa. Partnership for Maternal. Cape Town: Newborn and Child Health; 2006;32.

[16] Chaibva CN, Roos JH, Ehlers VJ. Adolescent mothers’ non-utilisation of antenatal care services in Bulawayo. Zimbabwe Curationis. 2009;32:14–21. 20225740 10.4102/curationis.v32i3.1219

[17] Mulinge N, Yusuf O, Aimakhu C. Factors influencing utilization of antenatal care services among teenage mothers in Malindi Sub-County Kenya-a cross sectional study. Sci J Publ Health. 2017;5:61–7.

[18] Ronen K, McGrath CJ, Langat AC, Kinuthia J, Omolo D, Singa B, et al. Gaps in adolescent engagement in antenatal care and prevention of mother-to-child HIV transmission services in Kenya. J Acquir Immune Defic Syndr. 2017;74:30. 10.1097/QAI.0000000000001176. 27599005 10.1097/QAI.0000000000001176 PMC5895459

[19] Ramraj T, Jackson D, Dinh T-H, Olorunju S, Lombard C, Sherman G, et al. Adolescent access to care and risk of early mother-to-child HIV transmission. J Adolesc Health. 2018;62:434–43. 10.1016/j.jadohealth.2017.10.007. 29269045 10.1016/j.jadohealth.2017.10.007 PMC6004498

[20] Kirsten I, Sewangi J, Kunz A, Dugange F, Ziske J, Jordan-Harder B, et al. Adherence to combination prophylaxis for prevention of mother-to-child-transmission of HIV in Tanzania. PLoS One. 2011;6:e21020. 10.1371/journal.pone.0021020. 21695214 10.1371/journal.pone.0021020 PMC3112206

[21] Gebrekristos LT, Groves AK, McNaughton Reyes L, Maman S, Moodley D. Ipv victimization in pregnancy increases postpartum STI incidence among adolescent mothers in Durban, South Africa. AIDS Care. 2020;32:193–7. 10.1080/09540121.2020.1742871. 32193964 10.1080/09540121.2020.1742871

[22] Groves AK, Gebrekristos LT, Reyes LM, Moodley D, Maman S. Describing relationship characteristics and postpartum HIV risk among adolescent, young adult, and adult women in South Africa. J Adolesc Health. 2020;67:123–6. 31992490 10.1016/j.jadohealth.2019.12.008 PMC7311245

[23] Wamoyi J, Stobeanau K, Bobrova N, Abramsky T, Watts C. Transactional sex and risk for HIV infection in sub-Saharan Africa: a systematic review and meta-analysis. J Int AIDS Soc. 2016;19:20992. 27809960 10.7448/IAS.19.1.20992 PMC5095351

[24] Li Y, Marshall CM, Rees HC, Nunez A, Ezeanolue EE, Ehiri JE. Intimate partner violence and HIV infection among women: a systematic review and meta-analysis. J Int AIDS Soc. 2014;17:18845. 10.7448/IAS.17.1.18845. 24560342 10.7448/IAS.17.1.18845 PMC3925800

[25] Groves AK, Bhushan NL, Stoner MCD, Gómez-Olivé FX, Kahn K, Pettifor AE. HIV and herpes simplex virus type 2 incidence among adolescent mothers in South africa: a longitudinal analysis of HIV prevention trials network 068 data. JAIDS J Acquir Immune Defic Syndr. 2022;89:e23-9. 10.1097/QAI.0000000000002872. 34855627 10.1097/QAI.0000000000002872 PMC8837695

[26] Cluver L, Rudgard WE, Toska E, Orkin M, Ibrahim M, Langwenya N, et al. Food security reduces multiple HIV infection risks for high-vulnerability adolescent mothers and non‐mothers in South Africa: a cross‐sectional study. J Int AIDS Soc. 2022;25:e25928. 10.1002/jia2.25928. 36008916 10.1002/jia2.25928 PMC9411725

[27] Govender D, Naidoo S, Taylor M. “My partner was not fond of using condoms and I was not on contraception”: understanding adolescent mothers’ perspectives of sexual risk behaviour in KwaZulu-Natal, South Africa. BMC Public Health. 2020;20:366. 10.1186/s12889-020-08474-2. 32197592 10.1186/s12889-020-08474-2 PMC7082996

[28] Bhushan N, Stoner MCD, Groves AK, Kahn K, Pettifor AE. Partnership dynamics and HIV-Related sexual behaviors among adolescent mothers in South africa: A longitudinal analysis of HIV prevention trials network 068 data n.d. 10.1016/j.jadohealth.2022.02.003 PMC9232891 35370076

[29] Connell RW, Cohen CR, Montandon M, Carrico AW, Shiboski S, Bostrom A, et al. Gender and power: society, the person, and sexual politics. Palo Alto: Stanford University Press; 1987.

[30] Closson K, Ndungu J, Beksinska M, Ogilvie G, Dietrich JJ, Gadermann A, et al. Gender, power, and health: measuring and assessing sexual relationship power equity among young sub-Saharan African women and men, a systematic review. Trauma Violence Abuse. 2022;23:920–37. 10.1177/1524838020979676. 33353490 10.1177/1524838020979676

[31] Nduna M. A magnifying glass and a fine-tooth comb: understanding girls’ and young women’s sexual vulnerability. Pretoria: CSA&G, Centre for Sexualities, AIDS and Gender, University of Pretoria; 2020.

[32] Govender D, Naidoo S, Taylor M. Prevalence and risk factors of repeat pregnancy among South African adolescent females. Afr J Reprod Health. 2019;23:73–87. 31034174 10.29063/ajrh2019/v23i1.8

[33] Kilburn K, Ranganathan M, Stoner MC, Hughes JP, MacPhail C, Agyei Y, et al. Transactional sex and incident HIV infection in a cohort of young women from rural South Africa. AIDS. 2018;32:1669. 29762176 10.1097/QAD.0000000000001866 PMC6082595

[34] Stern E, Buikema R. The relational dynamics of hegemonic masculinity among South African men and women in the context of HIV. Cult Health Sex. 2013;15:1040–54. 10.1080/13691058.2013.805817. 23805918 10.1080/13691058.2013.805817

[35] Leclerc-Madlala S. Age-disparate and intergenerational sex in Southern Africa: the dynamics of hypervulnerability. AIDS. 2008;22:S17. 10.1097/01.aids.0000341774.86500.53. 19033752 10.1097/01.aids.0000341774.86500.53

[36] Pulerwitz J, Mathur S, Woznica D. How empowered are girls/young women in their sexual relationships? Relationship power, HIV risk, and partner violence in Kenya. PLoS One. 2018;13:e0199733. 10.1371/journal.pone.0199733. 30024908 10.1371/journal.pone.0199733 PMC6053148

[37] Atkins K, Rucinski K, Mudavanhu M, Holmes L, Mutunga L, Kaufman MR, et al. Sexual relationship types, partner HIV self-testing, and pre-exposure prophylaxis among South African adolescent girls and young women: a latent class analysis. J Acquir Immune Defic Syndr. 2021;86:413–21. 10.1097/QAI.0000000000002569. 33196552 10.1097/QAI.0000000000002569 PMC10358829

[38] Stoner MCD, Nguyen N, Kilburn K, Gómez-Olivé FX, Edwards JK, Selin A, et al. Age-disparate partnerships and incident HIV infection in adolescent girls and young women in rural South africa: an HPTN 068 analysis. AIDS. 2019;33:83–91. 10.1097/QAD.0000000000002037. 30289813 10.1097/QAD.0000000000002037 PMC6279556

[39] Rousseau E, Wu L, Heffron R, Baeten JM, Celum CL, Travill D, et al. Association of sexual relationship power with PrEP persistence and other sexual health outcomes among adolescent and young women in Kenya and South Africa. Front Reprod Health. 2023. 10.3389/frph.2023.1073103. 37325240 10.3389/frph.2023.1073103 PMC10266091

[40] Bandiera O, Buehren N, Burgess R, Goldstein M, Gulesci S, Rasul I, et al. Empowering adolescent girls: evidence from a randomized control trial in Uganda. Washington, D.C.: World Bank; 2012.

[41] Maganja RK, Maman S, Groves A, Mbwambo JK. Skinning the goat and pulling the load: transactional sex among youth in Dar es Salaam, Tanzania. AIDS Care. 2007;19:974–81. 10.1080/09540120701294286. 17851993 10.1080/09540120701294286

[42] Selikow T-A, Ahmed N, Flisher AJ, Mathews C, Mukoma W. I am not ``umqwayito’’: a qualitative study of peer pressure and sexual risk behaviour among young adolescents in Cape Town, South Africa. Scand J Public Health. 2009;37:107–12. 10.1177/1403494809103903. 19493988 10.1177/1403494809103903

[43] Collins LM, Lanza ST. Latent class and latent transition analysis: with applications in the social, behavioral, and health sciences. Hoboken: Wiley; 2009.

[44] Vermunt JK, Magidson J. Latent class analysis. Sage Encyclopedia Social Sci Res Methods. 2004;2:549–53.

[45] Nguyen N, Powers KA, Miller WC, Howard AG, Halpern CT, Hughes JP, et al. Sexual partner types and incident HIV infection among rural South African adolescent girls and young women enrolled in HPTN 068: a latent class analysis. J Acquir Immune Defic Syndr. 2019;82:24–33. 10.1097/QAI.0000000000002096. 31169772 10.1097/QAI.0000000000002096 PMC6692200

[46] Khuzwayo N, Taylor M. Exploring the socio-ecological levels for prevention of sexual risk behaviours of the youth in uMgungundlovu District Municipality, KwaZulu-Natal. Afr j Prim Health Care Fam Med. 2018. 10.4102/phcfm.v10i1.1590. 10.4102/phcfm.v10i1.1590 PMC5843945 29781679

[47] Nalukwago J, Crutzen R, Borne B, van den, Bukuluki PM, Bufumbo L, Burke HM et al. Socio-cognitive factors associated with condom use, multiple sexual partnerships, and contraception use among sexually-active adolescent girls in Uganda 2018.

[48] Schaefer R, Gregson S, Eaton JW, Mugurungi O, Rhead R, Takaruza A, et al. Age-disparate relationships and HIV incidence in adolescent girls and young women: evidence from Zimbabwe. AIDS. 2017;31:1461–70. 10.1097/QAD.0000000000001506. 28426534 10.1097/QAD.0000000000001506 PMC5457819

[49] Thior I, Rowley E, Mavhu W, Kruse-Levy N, Messner L, Falconer-Stout ZJ, et al. Urban-rural disparity in sociodemographic characteristics and sexual behaviors of HIV-positive adolescent girls and young women and their perspectives on their male sexual partners: a cross-sectional study in Zimbabwe. PLoS One. 2020;15:e0230823. 10.1371/journal.pone.0230823. 32324764 10.1371/journal.pone.0230823 PMC7179911

[50] Brown LJ, Reddy T, Mannell J, Burgess R, Shai N, Washington L, et al. A latent class analysis of young women’s co-occurring health risks in urban informal settlements in Durban, South Africa. SSM - Mental Health. 2023;4:100273. 10.1016/j.ssmmh.2023.100273.

[51] Government of the Kingdom of Eswatini. Swaziland HIV incidence measurement survey 2 (SHIMS2) 2016–2017. Final report. Mbabane: Government of the Kingdom of Eswatini; 2019.

[52] Ministry of Health. Lesotho, centers for disease control and prevention (CDC), and ICAP at Columbia, university. Lesotho Population-based HIV impact assessment (LePHIA) 2016–2017: final Report. Maseru, Lesotho, Atlanta, Georgia, and New York: Ministry of Health, CDC, and ICAP; 2019.

[53] Ministry of Health, Malawi. Malawi Population-Based HIV Impact Assessment (MPHIA) 2015–2016: Final Report. Lilongwe, Ministry of Health: 2018.

[54] Ministry of Health and Social Services (MoHSS). Namibia Population-based HIV Impact Assessment (NAMPHIA) 2017: Final Report. Windhoek: MoHSS; 2019.

[55] Tanzania Commission for AIDS (TACAIDS). Zanzibar AIDS commission (ZAC). Tanzania HIV impact survey (THIS) 2016–2017: final report. Tanzania.: Dar Es Salaam; 2018.

[56] Ministry of Health, Uganda. Uganda Population-based HIV Impact Assessment (UPHIA) 2016–2017: Final Report. Kampala: Ministry of Health; 2019.

[57] Ministry of Health. Zambia Population-based HIV Impact Assessment (ZAMPHIA) 2016: Final Report. Lusaka: Ministry of Health; 2019.

[58] Ministry of Health and Child Care (MOHCC), Zimbabwe. Zimbabwe Population-based HIV Impact Assessment (ZIMPHIA) 2015–2016: Final Report. Harare: MOHCC; 2019.

[59] Karim SSA, Baxter C. HIV incidence rates in adolescent girls and young women in sub-Saharan Africa. Lancet Glob Health. 2019;7:e1470. 10.1016/S2214-109X(19)30404-8. 31607449 10.1016/S2214-109X(19)30404-8

[60] Neal S, Channon AA, Chandra-Mouli V, Madise N. Trends in adolescent first births in sub-Saharan Africa: a tale of increasing inequity? Int J Equity Health. 2020;19:1–11. 10.1186/s12939-020-01251-y PMC7487507 32887618

[61] Shisana O, Rehle T, Simbayi LC, Zuma K, Jooste S, Zungu N et al. South African national HIV prevalence, incidence and behaviour survey, 2012 2014. 10.2989/16085906.2016.1153491 27002359

[62] Population-based HIV Impact Assessment (PHIA) Data use manual. NY: 2021.

[63] Groves AK, Reyes HLM, Gebrekristos LT, Moodley D, Maman S. Examining why age-disparate relationships influence unsafe sex postpartum among South African women: relationship control and physical partner violence as explanatory mechanisms. J Interpers Violence. 2020. 10.1177/0886260520944531. 32748693 10.1177/0886260520944531

[64] Harling G, Newell M-L, Tanser F, Kawachi I, Subramanian SV, Bärnighausen T. Do age-disparate relationships drive HIV incidence in young women? Evidence from a population cohort in rural KwaZulu-Natal, South Africa. JAIDS J Acquir Immune Defic Syndr. 2014;66:443. 10.1097/QAI.0000000000000198. 24815854 10.1097/QAI.0000000000000198 PMC4097949

[65] Bajunirwe F, Semakula D, Izudi J. Risk of HIV infection among adolescent girls and young women in age-disparate relationships in sub-Saharan Africa. AIDS. 2020;34:1539–48. 10.1097/QAD.0000000000002582. 32443063 10.1097/QAD.0000000000002582

[66] Stoner MCD, Edwards JK, Miller WC, Aiello AE, Halpern CT, Julien A, et al. Effect of Schooling on Age-Disparate Relationships and Number of Sexual Partners Among Young Women in Rural South Africa Enrolled in HPTN 068. J Acquir Immune Defic Syndr. 2017;76:e107–14. 10.1097/QAI.0000000000001544. 28902703 10.1097/QAI.0000000000001544 PMC5680112

[67] Stoebenau K, Heise L, Wamoyi J, Bobrova N. Revisiting the understanding of transactional sex in sub-Saharan Africa: a review and synthesis of the literature. Soc Sci Med. 2016;168:186–97. 10.1016/j.socscimed.2016.09.023. 27665064 10.1016/j.socscimed.2016.09.023

[68] Vermunt JK. Latent class modeling with covariates: two improved three-step approaches. Polit Anal. 2010;18:450–69.

[69] Henry KL, Muthén B. Multilevel latent class analysis: an application of adolescent smoking typologies with individual and contextual predictors. Struct Equ Model Multidiscip J. 2010;17:193–215. 10.1080/10705511003659342. 10.1080/10705511003659342 PMC2968712 21057651

[70] Bakk Z, Tekle FB, Vermunt JK. Estimating the association between latent class membership and external variables using bias-adjusted three-step approaches. Sociol Methodol.2013;43:272–311. 10.1177/0081175012470644.

[71] Muthén LK, Muthén BO. Mplus user’s guide. 8th ed. Los Angeles: Muthén & Muthén; 2017.

[72] Vermunt JK. Multilevel latent class models. Sociol Methodol. 2003;33:213–39.

[73] Lukočienė O, Varriale R, Vermunt JK. The simultaneous decision(s) about the number of lower- and higher-level classes in multilevel latent class analysis. Sociol Methodol. 2010;40:247–83. 10.1111/j.1467-9531.2010.01231.x.

[74] Core Team R. A language and environment for statistical computing. Vienna: R Foundation for Statistical Computing; 2021.

[75] Nkwemu S, Jacobs CN, Mweemba O, Sharma A, Zulu JM. They say that i have lost my integrity by breaking my virginity”: experiences of teen school going mothers in two schools in Lusaka Zambia. BMC Public Health. 2019. 10.1186/s12889-019-6394-0. 30642304 10.1186/s12889-019-6394-0 PMC6332585

[76] Ajayi AI, Akpan W, Goon DT, Nwokocha EE, Adeniyi OV. Tough love : socio-cultural explanations for deadly abortion choices among Nigerian undergraduate students. Afr J Phys Act Health Sci (AJPHES). 2016;22:711–24. 10.10520/EJC195990.

[77] Schaffnit SB, Wamoyi J, Urassa M, Dardoumpa M, Lawson DW. When marriage is the best available option: perceptions of opportunity and risk in female adolescence in Tanzania. Glob Public Health. 2021;16:1820–33. 10.1080/17441692.2020.1837911. 33131404 10.1080/17441692.2020.1837911

[78] Madhavan S, Harrison A, Sennott C. Management of non-marital fertility in two South African communities. Cult Health Sex. 2013;15:614–28. 10.1080/13691058.2013.777475. 23600721 10.1080/13691058.2013.777475 PMC3674186

[79] Evans M, Risher K, Zungu N, Shisana O, Moyo S, Celentano DD, et al. Age-disparate sex and HIV risk for young women from 2002 to 2012 in South Africa. J Int AIDS Soc. 2016;19:21310. 28364564 10.7448/IAS.19.1.21310 PMC5384594

[80] Hampanda KM, Pelowich K, Freeborn K, Graybill LA, Mutale W, Jones KR, et al. Strategies to increase couples HIV testing and counselling in sub-Saharan Africa: a systematic review. J Int AIDS Soc. 2023. 10.1002/jia2.26075. 36929284 10.1002/jia2.26075 PMC10020817

[81] Stoner MCD, Edwards JK, Miller WC, Aiello AE, Halpern CT, Julien A, et al. Does partner selection mediate the relationship between school attendance and HIV/Herpes simplex virus-2 among adolescent girls and young women in South Africa: an analysis of HIV prevention trials network 068 data. JAIDS J Acquir Immune Defic Syndr. 2018;79:20. 10.1097/QAI.0000000000001766. 29847479 10.1097/QAI.0000000000001766 PMC6092209

[82] Stoner MC, Pettifor A, Edwards JK, Aiello AE, Halpern CT, Julien A, et al. The effect of school attendance and school dropout on incident HIV and HSV-2 among young women in rural South Africa enrolled in HPTN 068. AIDS. 2017;31:2127–34. 10.1097/QAD.0000000000001584. 28692544 10.1097/QAD.0000000000001584 PMC5599334

[83] Gubi D, Nansubuga E, Wandera SO. Correlates of intimate partner violence among married women in Uganda: a cross-sectional survey. BMC Public Health. 2020;20:1–11. 32586297 10.1186/s12889-020-09123-4 PMC7318470

[84] Dunkle KL, Jewkes RK, Brown HC, Gray GE, McIntryre JA, Harlow SD. Gender-based violence, relationship power, and risk of HIV infection in women attending antenatal clinics in South Africa. Lancet. 2004;363:1415–21. 10.1016/S0140-6736(04)16098-4. 15121402 10.1016/S0140-6736(04)16098-4

