# Supplementary material for: Sexual Relationship Typologies, Multilevel Determinants, and HIV Among Adolescent Mothers in Eastern and Southern Africa: A Multilevel Latent Class Analysis
Source: AIDS Behav. 2026 Jan 16;30(7):1996–2011. doi: 10.1007/s10461-025-05024-y (PMC13400485; doi:10.1007/s10461-025-05024-y)
Supplement: Supplementary file 1 — Supplementary Material 1 [file 10461_2025_5024_MOESM1_ESM.docx]

# Table 1 shows the measurement of relationship characteristics used to identify relationship typologies.

# Table 2 shows the variance inflation factors (VIF) among the individual and community-level measures. All VIF are <10 and therefore multicollinearity is not a concern for our analyses.

# Table 3 shows the fit indices for the multilevel latent class analyses. Model 1 determines the number of classes at the community-level (when there are 3 classes at the individual-level) and Model 2 confirms the number of classes at the individual-level (when there are 2 classes community-level).

# **Table 1. Measurement of relationship characteristics**

| **Relationship Characteristics** | **Survey question and response options** | **Coding for models** |
| --- | --- | --- |
|  |  |  |
| Age disparate relationship | How old is [Partner’s Name]?  [ ] years | Partner ≥ 5 years older than adolescent mother  Partner < 5 years older than adolescent mother |
| Marital status | What is your marital status now: are you married, living together with someone as if married, widowed, divorced, or separated?  1 - Married  2 - Living together  3 - Widowed  4 - Divorced  5 - Separated | Married or cohabiting  Not married or cohabiting |
| Reported transactional sex | Did you enter into a sexual relationship  with [Partner’s Name] because [Partner’s Name] provided you with or you expected that [Partner’s Name] would provide you with material support  or help you in other ways?  1 - Yes  2 - No | Yes  No |
| Uninformed of partner’s HIV status | What is the HIV status of [Partner’s Name]?  1 - I think he/she is positive, or he/she told me he/she is positive, or he/she is positive, tested together  2 - I think he/she is negative  3 - He/she told me he/she is negative  4 - He/she is negative, tested together  5 - Don't know status | Yes (responded I think he/she is negative or don't know status)  No (all other responses) |
| Worked in the past year | Have you done any work in the last 12  months for which you received a paycheck, cash or goods as payment?  1 - Yes  2 - No | Yes  No |

# **Table 2. Variance Inflation Factors for individual and community-level measures**

| **Measures** | **Variance Inflation Factors** |
| --- | --- |
|  |  |
| **Individual-level** |  |
| Age | 1.0 |
| Schooling | 1.0 |
| Low wealth household | 1.5 |
| Number of partners | 1.0 |
| **Community-level** |  |
| Urbanicity | 1.3 |
| Proportion of households with low wealth | 1.8 |

### **Table 3. Fit Indices for Multilevel Latent Class Analysis (MLCA)**

|  | **Level-2 classes** | | |  |
| --- | --- | --- | --- | --- |
| **Model 1. MLCA 3 classes at Level-1** | 2 | 3 | 4 |  |
| Log likelihood | -8,692.63 | -8,657.81 | -8,631.08 |  |
| AIC | 17,455.25 | 17,421.61 | 17,404.16 |  |
| BIC | 17,662.57 | 17,735.55 | 17,824.72 |  |
| ssBIC | 17,551.36 | 17,567.15 | 17,599.13 |  |
|  | **Level-1 classes** | | | |
| **Model 2. MLCA 2 classes at Level-2** | 2 | 3 | 4 | 5 |
| Log likelihood | -8,999.7 | -8,974.03 | -8,957.55 | -8,946.82 |
| AIC | 18,045.4 | 18,018.06 | 18,009.1 | 18,011.64 |
| BIC | 18,181.64 | 18,225.38 | 18,287.5 | 18,361.12 |
| ssBIC | 18,108.56 | 18,114.17 | 18,138.17 | 18,173.66 |
| *AIC=Akaike Information Criterion; BIC=Bayesian Information Criterion; ssBIC=sample sized adjusted Bayesian Information Criterion; BLRT= Bootstrapped Likelihood Ratio Rest* | | | | |
